# Genome-wide SNP analyses reveal population structure of *Portunus pelagicus* along Vietnam coastline

**DOI:** 10.1371/journal.pone.0224473

**Published:** 2019-11-05

**Authors:** Binh Thuy Dang, Muhammad Arifur Rahman, Sang Quang Tran, Henrik Glenner

**Affiliations:** 1 Department of Biology, Institute for Biotechnology and Environment, Nha Trang University, Nha Trang City, Vietnam; 2 Department of Graduate Studies, Nha Trang University, Nha Trang City, Vietnam; 3 Department of Biological Sciences, University of Bergen, Bergen, Norway; University of Iceland, ICELAND

## Abstract

The blue swimming crab (*Portunus pelagicus* Linnaeus, 1758) is one of the commercially exploited crab fishery resources in Vietnam. This is the first study to provide a broad survey of genetic diversity, population structure and migration patterns of *P*. *pelagicus* along the Vietnamese coastline. The crab samples were collected from northern, central and southern Vietnam. Here, we used a panel of single nucleotide polymorphisms (SNPs) generated from restriction site-associated DNA sequencing (RADseq). After removing 32 outlier loci, 306 putatively neutral SNPs from 96 individuals were used to assess fine-scale population structure of blue swimming crab. The mean observed heterozygosity (Ho) and expected heterozygosity (He) per locus was 0.196 and 0.223, respectively. Pairwise *Fst* and hierarchical AMOVA supported significant differentiation of central and northern from southern populations (P<0.01). Population structure analyses revealed that *P*. *pelagicus* in the south is a separate fisheries unit from the north and center. Contemporary migration patterns supported high migration between northern and central populations and restricted genetic exchange within the southern population. In contrast, historic gene flow provides strong evidence for single panmictic population. The results are useful for understanding current status of *P*. *pelagicus* in the wild under an environment changing due to natural and anthropogenic stresses, with implications for fisheries management.

## Introduction

The tropical to subtropical Vietnamese coastal zone is divided into the Gulf of Tonkin in the North, the central coast, the southeast coast and the Gulf of Thailand in the South [1,2]. The exclusive economic zone (EEZ) covers about 1 million km^2^ and 3260 km of coastline along the East Sea (the Vietnamese name for the South China Sea). In winter, currents flow in a North East-South West direction while in summer, ocean currents flow from the South West-North East [3,4] with the eddies existing at the southern and central parts of the Vietnamese coastline [3]. Climate change and human activities including aquaculture, overexploitation, and illegal fishing are threatening coastal habitats (e.g. seagrass beds) and biodiversity [1,5–7].

The blue swimming crab (*Portunus pelagicus*) is a scavenging tropical marine species, widely distributed in the Indian and Pacific oceans, the East coast of Africa, the Mediterranean Sea and southern Japan [7–10]. In Vietnam, it is distributed in the wild throughout the long coastline and aggregated densely in Kien Giang (south of the Mekong Delta) waters [8,11]. It matures and reproduces continuously in one spawning season [12–14]. Planktonic larvae may be transported long distances, supposedly driven by a combination of factors such as temperature, wind, surface currents and salinity [14–17], and spatial distribution depends on larval stages [15,16,18].

*P*. *pelagicus* is present in large numbers with great value for commercial fisheries exporting to the USA, Europe and Japan [7,19,20]. According to the FAO (2016) [21], global catch and aquaculture production were 265,896 tonnes, and 29 tonnes, respectively. In Vietnam, the total catch in 2010 was 11,300 tonnes, while production in Kien Giang reached 7,800 tonnes in 2013, suggesting a decline due to overharvesting [11]. Gillnet and crab traps were reported as the dominant fishing gears of *P*. *pelagicus* (accounting for 77.8% and 22.2%, respectively) [11].

A crab management plan for Vietnam is in place. However, due to unsystematic application of management measures (the minimum landing size and the closed season), and lack of demographic information, management is considered ineffective [22,23]. Recently, genetic studies have increasingly been applied to improve understanding of stock size, gene flow, distribution and migration patterns of subpopulations in mixed fisheries [24–27]. Population information including connectivity across species distribution range, exchange rate and source-sink dynamics are important for understanding potential impacts of bio-physical factors [28–31], human-induced fragmentation [32] and pollution [33,34], or overexploitation [35–37]. Among the wide-range of molecular approaches, restriction site-associated DNA sequencing (RADseq) is well known for its ability to identify and score thousands of single nucleotide polymorphisms (SNPs), which are randomly distributed across the target genomes using next generation sequencing [38–40]. RAD methods are being used and developed with many techniques such as mbRAD [41], 2b-RAD [42], ddRAD [38] and ezRAD [43].

Several studies have revealed different population structuring of *P*. *pelagicus* throughout its distribution range. In the early 2000s, Yap et al. (2002) [44] and Sezmis et al (2004) [45] detected high population genetic structure in Australia with microsatellites. Similarity, Klinbunga (2007, 2010) [46,47] using DNA polymorphism assays (RADPs and AFLPs) identified strong genetic population structure in Thailand. A more recent studies utilizing mitochondrial DNA markers discovered either limited or high genetic structure in China and the Philippines, respectively [48,49]. In both studies, cryptic species of *P*. *pelagicus* have been reported as previously recorded by Lai et al (2010 [10]. Additionally, using microsatellites, Chai et al (2016) [50] identified low genetic structure of *P*. *pelagicus* in Malaysia, while Ren et al. (2016) [51] found distinct populations in Indonesia with RADP plus nuclear DNA marker (16S rDNA). Recently, Miao et al. (2017) [52] applied RADseq to investigate 91 SNPs suggesting these as helpful makers for population research resources of this valuable species. Despite the economic and ecological importance, no studies are known of population genetics of *P*. *pelagicus* in Vietnam, although limited published studies examining genetic structure of marine organisms have indicated high connectivity in the dynamic and complex Vietnam East Sea waters [29,53].

The goal of this study was to develop SNPs using RADSeq, previously not accomplished for *P*. *pelagicus* in Vietnam, to better understand fine-scale population structuring and gene flow along the Vietnamese coastline and to provide data on resilience and sustainability for fisheries management.

## Materials and methods

### Sampling sites and tissue collection

**Fig 1:**
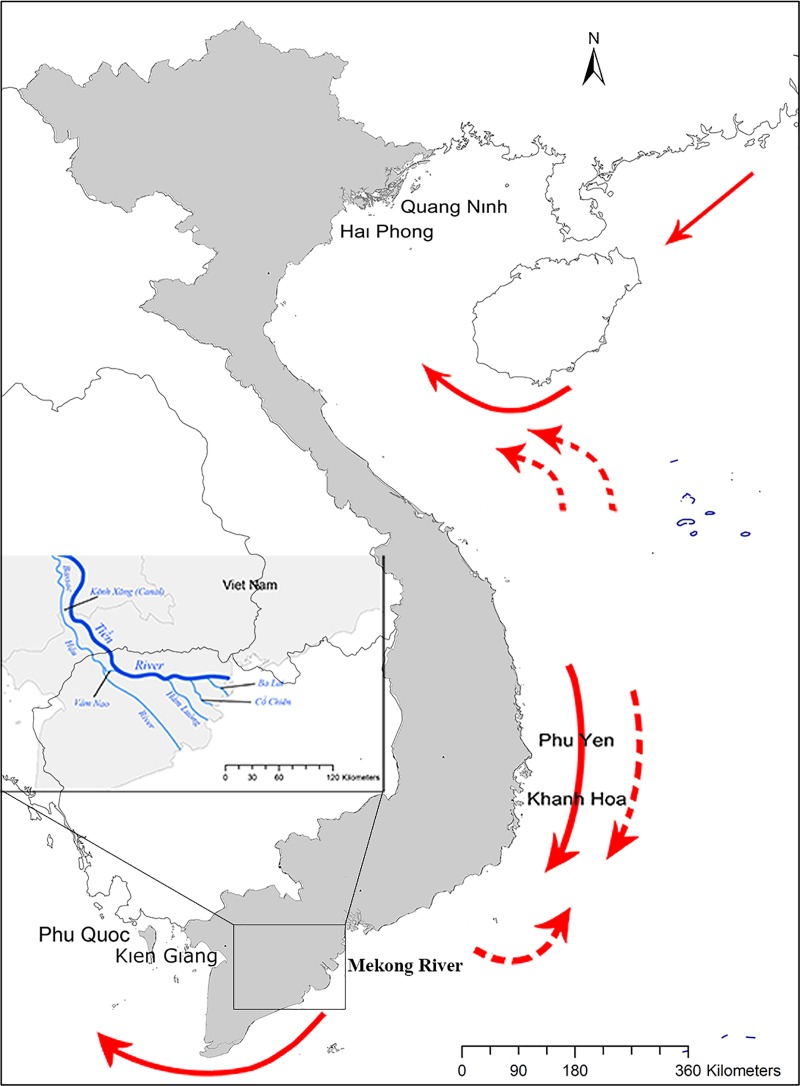
**Sampling map of *Portunus pelagicus* and surface currents following northeast (bold line) and southwest (dash line) monsoons**, INSET: Mekong River (black box) in Mekong delta, Vietnam.

Blue swimming crabs were collected along the north-south geographical temperature gradient: Cat Ba Island—Hai Phong City; Ha Long Bay—Quang Ninh Province (northern population), Nha Trang Bay and Van Phong Bay—Khanh Hoa province; Song Cau and Tuy Hoa—Phu Yen province (central population), and Phu Quoc Island, Rach Gia City—Kien Giang Province (southern population) (**Fig 1, Table 1**).

**Table 1:** *Portunus pelagicus* sample site information and genetic diversity. Number of individuals successfully genotyped and used in analyses (Nse), observed number of alleles (Na), effective number of alleles (Ne), observed (Ho) and expected (He) heterozygosity, percentage of polymorphic loci (%P) and the inbreeding coefficient (G_IS_).

Pop ID	Sampling site	Nse	Na	Ne	Ho	He	G_IS_	%P
Northern	Quang Ninh	16	1.923	1.315	0.166	0.211	0.185	92.31
	Hai Phong	24						
Central	Phu Yen	19	1.982	1.378	0.207	0.246	0.154	98.22
	Khanh Hoa	11						
Southern	Phu Quoc	16	1.885	1.370	0.216	0.233	0.265	88.46
	Rach Gia	10						
	**Total/Mean**	**96**	**1.930**	**1.354**	**0.196**	**0.230**	**0.168**	**93.00**

The crabs were collected at the exploitation sites, transported alive in aerated sea water to the laboratory where they were kept in aquaria until tissue sampling. Information on sampling sites and crab size (carapace width and weight) were presented in **S1 Table**. All tissue samples were taken from chelipeds of fresh crab and preserved in 95% ethanol.

### Research methodology

#### DNA extraction and digestion

Genomic DNA was extracted from preserved tissue samples using the DNeasy Blood & Tissue Kit (Qiagen) following the manufacturer's instructions, and treated with RNase (100 mg/mL) to remove residual RNA. Extracted DNA was eluted three times (100 μl elution/time) to get better DNA quality. All elutions were assessed using gel electrophoresis (1% agarose gel). The best elution (sharp, high weight molecular bands, no smear) was selected to determine the concentration by Qubit^®^ 2.0 Fluorometer (Invitrogen). Selected DNA templates (100 ng, concentration ≥ 3 ng/μl) were then purified using AMPureXP (Agencourt) beads using a 2:1 template to bead volume ratio with the beads left in.

Purified DNA from each crab individual was simultaneously digested with two restriction enzymes: MboI and Sau3AI (NEB). Each digestion was performed in 25 μl reactions: 2.5 μl SmartCut Buffer (10X), 0.5 μl MboI and 0.5 μl Sau3AI (5 unit/μl), and 21.5 μl of DNA template (eluate from the beads). Digestions were incubated at 37°C for 3 h to overnight, and then 65°C for 20 min, cleaned with PEG solution (10 g PEG, 7.3 g NaCl, plus water up to 49 ml), and eluted with 20.1 μl Illumina Resuspension Buffer.

#### EzRAD library preparation

Cleaned digestions were inserted directly into the Illumina TruSeq nano DNA library Prep kit following the Sample Preparation v2 Guide starting with the “Perform End Repair” step for one-third volume reactions (Supplement S1 [38]). Digested libraries were end-repaired, 350 bp size-selected by SP bead. Firstly, SP bead:H20 (1.5:1) were added to removed >550 bp fragments, the supernatant collected and applied to 10 μl SP bead to subsequently remove <350 bp fragments”. The 3ʹ ends of selected libraries were then adenylated and Illumina adapters were ligated to the digested genomic DNA samples. PCR reactions were performed using a total volume of 15 μl including 1.5 μl Illumina PCR Primer Cocktail, 6 μl Illumina Enhanced PCR Mix, 1.875 μl ddH_2_O and 5.625 μl DNA libraries. Biorad thermocyclers (Icycler) were used under the following temperature program: initial denaturation at 95°C for 3 min, followed by 8 cycles of 98°C for 20s, 60°C for 15s and 72°C for 30s. Final extension was done at 72°C for 5 min and the soaking temperature was set to 4°C.

PCR products (The 400–500 bp fragments of which 120 bp are the ligated adapters) were inspected using a 1.5% agarose gel with ethidium bromide and bands were visualized under UV transilluminator. PCR products were purified using SP Beads (1:1), and quantified using qPCR. DNA libraries were sequenced as paired-end 100 bp runs on HiSeq 2500/4000 system (Illumina) in Texas A&M University Corpus Christi Genomics Core Laboratory, USA.

### Data analyses

#### SNPs discovery and filtering

SNP detection was implemented by dDocent v2.0 pipeline [54]. At first, raw FastQ files were trimmed using Trimmomatic v0.3 [55] to simultaneously remove Illumina adapter sequences, and any bases that had a quality score (Q-score) of less than 10 [43]. These reads were clustered and input into *de novo* reference assembly in Rainbow v2.0.2 [56] and CD-HIT v4.6.1 [57,58] based on overall sequence similarity (90% by default). Quality-trimmed reads were mapped to the reference using BWA v0.7.12 [59,60] with the MEM algorithm [61]. SAM files were converted to BAM files using SAMTOOLS [62] and output was further restricted to reads with mapping quality above 10.

SNP calling was performed using Freebayes v0.9.21 [63] with default parameters. Raw SNP files were concatenated into a single variant call format (VCF) file using VCFtools v0.1.11 [64]. The raw SNPs were then filtered with VCFtools and VCFfilter. Primary filtering steps included: minor allele frequency (MAF > 0.05), minimum mean depth (≥ 5 mean DP *≤* 10), INDEL loci (this step decomposed insertion and deletion genotypes), Hardy-Weinberg Equilibrium (HWE with p < 0.001), mean quality score (*Q* > 30), max-missing (to apply a genotype call rate of 90% across all individuals), and number of variants (restricted to bi-allelic SNPs). Secondary filtering steps included keeping loci based on allelic balance (AB > 0.3), mean mapping quality (0.9 < MQM/MQMR < 1.05), and proportion of alternate alleles (0.05 < PAIRED/PAIREDR < 1.75). Putative SNPs were submitted to rad_haplotyper (https://github.com/chollenbeck/rad_haplotyper) to remove possible paralogs, and one SNP filtering to get the validated SNP panel.

#### Outlier loci detection and Linkage-disequilibrium (LD) analysis

Our final filtered panel of SNPs was run in BayeScan v2.1 [65] under default parameter settings to identify loci under divergent or balancing selection. A false discovery rate (FDR) correction of 0.05 was applied [66].

LD was measured as the squared pairwise correlation coefficient between loci (r^2^) calculated using the ‘LD’ function in the R package ‘genetics’[67]. Selected outlier clusters (SOC) and Compound outlier clusters (COC) were identified by LD network analysis using R package ‘LDna’ [68], optimal value of φ and |E|min parameter and LD threshold was set up for SOC. LD network were constructed using the R package ‘igraph’ [69].

All loci putatively identified by either programs were removed from the dataset to generate a panel of neutral SNPs.

#### Genetic diversity and relatedness

Numbers of alleles (N_a_), effective numbers of alleles (N_e_), expected (*H*_*e*_) and observed (*H*_*o*_) heterozygosity, and inbreeding coefficients (*G*_IS_) were calculated for each sampled population and over all populations across the Vietnam coastline using GenAlexv6.5 [70] and GenoDive v.2.0b27 [71].

High levels of relatedness can impact analyses of population structure and estimates of population size, so relationships between individuals were estimated with the R package ‘related’ [72] using the dyadic ([73] and triadic [74] maximum likelihood estimators and allowing for inbreeding. For both estimators 95% confidence intervals were calculated with 500 bootstrap events for each pairwise comparison. Potential pairs were identified as exhibiting a related value. Due to the imbalanced numbers of related pairs among populations leading to reduced sample size and avoiding positive bias in estimates due to underestimating relatedness in the overall population [75], further analyses were run with two datasets, one containing all individuals (with related pairs) and one with one putative individual removed per related pair (related individuals removed).

#### Analyses of population structure

Pairwise comparisons of *F*st values between *P*. *pelagicus* populations werecomputed in ARLEQUIN [76] to test for significant differentiation among sampled sites. All p-values underwent FDR correction to avoid false positives resulting from multiple comparisons [66]. A hierarchical analysis of molecular variance (AMOVA) was performed to test for significant population structure within species, following two group options: geographically-defined populations (northern, central and southern) and combined individuals from the north and center into a single population, and considering individuals from the south as a separate population (northern-central and southern) using the program ARLEQUIN.

We tested for population connectivity and structure in the program Structure v2.3.4 [77,78] using a model-based Bayesian clustering method to infer the number of lineages, *K*, in a dataset. Structure was run to test *K* values of 1 through 4 with 10,000 iterations of burn-in followed by 5,000 Markov Chain Monte Carlo (MCMC) steps, using the correlated allele frequencies admixture model. The optimal value of *K* was evaluated using the Evanno method [79] by Structure Harvester v0.6.94 [80]. A Discriminant analysis of principal components (DAPC) was performed using the R package ‘adegenet’ [81]. This analysis provides a graphic description of the genetic divergence among populations in multivariate space.

#### Migration patterns

Historic gene flow between populations was estimated using the Bayesian inference implemented in MIGRATE-n v3.6.11 [82]. MIGRATE-n’s implementation of coalescent theory measures migration 4 × *Ne* generations in the past [32,83]. Sample sizes were reduced for each population to obtain 200 loci genotyped in 100% of individuals used for the analysis. The run was performed using 500,000 recorded genealogies sampled every 100 steps, preceded by a burn-in of 20,000. Four hot chains were used with temperatures: T1 = 1.0, T2 = 1.5, T3 = 3.0 and T4 = 1.0x10^6^. After optimization, the maximum mutation-scaled effective population size (*θ*) prior was set at 0.1 while the maximum mutation-scaled migration (*M*) prior was set at 20,000. Five hypotheses of migration among populations were tested: (1) symmetric migration rates between all sites (Panmixia Model), (2) non-symmetric migration rates between all sites (Full Model) (3) migration between all sites only from the north to the south (North-South Model), (4) migration between all sites only from the south to the north (South-North Model), (5) migration occurring only between neighboring, north-center sites but no migration between south population (South Separate Model). The most likely model was chosen using the Bezier ln produced by Migrate-N according to Beerli et al. (2009) [84]. To elucidate the recent migration patterns, estimate relative migration levels (Nm) between populations were calculated based on neutral SNPs using divMigrate function [85] of R package “diveRsity” [86]. Gene flow patterns were visualized using network graphics produced using the R package “qgraph” [87].

Ethics Statement: All crab were collected from fish markets or through normal fishing activities and therefore within the guidelines of approved IACUC procedures, and did not need sampling permission in Vietnam. This study did not involve protected or endangered species

Data Archiving: Upon acceptance, the unmodified sequence data in FASTQ format used in this research along with corresponding metadata will be uploaded and archived in the publicly accessible Genomic Observatories Metadatabase (GeOMe, http://www.geome-db.org/).

## Results

### SNP discovery and filtering

Results of 165 libraries of *P*. *pelagicus* along the Vietnamese coastline generated 604123297 reads with a reading length of 101 bp. The optimal reference assembly of 3280843 bp was constructed from 9583 RAD tags. Initially, 107115 raw SNPs were detected. After filtering steps, 96 individuals were successfully genotyped at 338 valid SNPs. Information on individuals removed and SNPs retained at each step of filtering and data analysis is presented in **S2 Table.**

### Outlier loci detection

**Fig 2:**
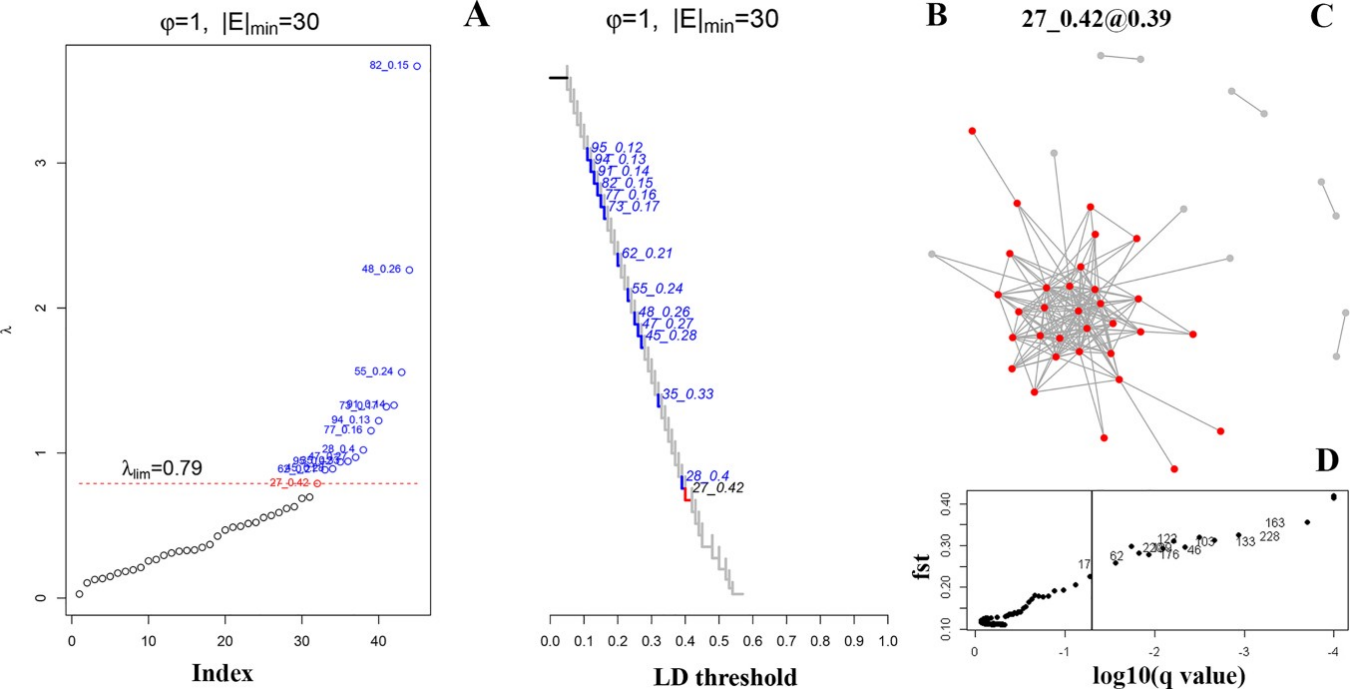
LD network analysis and outlier test results of *Portunus pelagicus*. (A) All λ values in increasing order with values above λ_min_ corresponding to outlier clusters. Parameter values for φ and |E|min are shown above plots. (B) A clustering tree of pairwise r^2^ values from putative 338 SNPs. Branches corresponding to SOCs and COCs are indicated in red and blue, respectively. (C) Selected SOC is shown at an LD threshold where it is joined by a single link to other loci. (D) Results of Bayesian outlier test, locus specific Fst coefficient is plotted against log10 (q value) for the model including selection, the vertical line represents a false discovery threshold of 0.05.

BayeScan identified thirteen SNPs as outliers (q<0.05, α>0, FDR ≤ 0.05) from the panel of 338 putative SNPs used to detect selection footprints (**Fig 2D**). LD network presented one selected outlier cluster (SOC) including 32 loci (φ = 1 and |E|_min_ = 30, λ_min_ = 0.79, LD threshold = 0.39) (**Fig 2A–2C**). The outlier loci detected by BayeScan were included in the SOC of LD network. In total, 32 loci were removed from the SNP panel and the 306 remaining loci were assumed to be neutral.

### Genetic diversity and relatedness

Genetic diversity of *P*. *pelagicus* is presented in **Table 1**. The mean observed number of alleles (Na) and effective number of alleles (Ne) of the populations were 1.930 and 1.354 respectively. Average observed (Ho) and expected heterozygosity (He) were shown across all populations, ranging from 0.166–0.216 (mean 0.196) to 0.211–0.246 (mean 0.23), respectively. Inbreeding coefficients ranged from 0.154 (Center) to 0.265 (South), with an overall G_IS_ for all individuals at 0.168. % Polymorphic sites were highest in the central (98.22%), and lowest in the southern population (88.46%).

Analyses of genetic relationships between individuals revealed 23 pairs of putative half siblings and 5 pairs of putative full sibling (**Table 2**) following removal of 16 individuals (**S2 Table**). Both full and half siblings (22 pairs) occurred abundantly within the southern population, while the other sibling pairs occurred in remaining sampling sites (**Table 2**).

**Table 2:** Results of relatedness analysis for two estimators calculated with *related* for pairs of putative siblings. Coefficients of relatedness (r) with 95% confidence intervals in parentheses are presented for both the Dyadml likelihood estimator and the trioml likelihood estimator. The most likely relationship for each pair is also shown.

Specimen Pairs	Groupings	Trioml (CI 95%)	Dyadml (CI 95%)	Relationship
KG104/KG119	KGKG	0.303 (0.202–0.413)	0.303 (0.201–0.42)	Half siblings
KG102/KG112	KGKG	0.302 (0.164–0.560)	0.307 (0.201–0.592)	Half siblings
KG104/KG117	KGKG	0.303 (0.207–0.432)	0.309 (0.203–0.429)	Half siblings
KG105/KG118	KGKG	0.310 (0.194–0.380)	0.311 (0.204–0.385)	Half siblings
KG109/KG119	KGKG	0.311(0.206–0.421)	0.311 (0.205–0.420)	Half siblings
KG102/KG110	KGKG	0.306 (0.205–0.407)	0.313 (0.205–0.408)	Half siblings
KG104/KG116	KGKG	0.308 (0.120–0.365)	0.314 (0.206–0.371)	Half siblings
KG103/KG110	KGKG	0.314 (0.203–0.397)	0.322 (0.206–0.406)	Half siblings
QN206/QN213	QNQN	0.323 (0.206–0.414)	0.323 (0.207–0.418)	Half siblings
QN213/HP207	QNHP	0.323 (0.202–0.447)	0.327 (0.209–0.446)	Half siblings
PY111/QN214	PYQN	0.311 (0.214–0.420)	0.332 (0.214–0.420)	Half siblings
KG102/KG109	KGKG	0.325 (0.222–0.435)	0.332 (0.229–0.436)	Half siblings
KG101/KG109	KGKG	0.322 (0.245–0.433)	0.333 (0.230–0.436)	Half siblings
KG103/KG104	KGKG	0.327 (0.232–0.450)	0.334 (0.231–0.449)	Half siblings
KG109/KG117	KGKG	0.313 (0.234–0.483)	0.336 (0.234–0.483)	Half siblings
KG104/KG105	KGKG	0.336 (0.238–0.426)	0.342 (0.237–0.436)	Half siblings
KG103/KG106	KGKG	0.333(0.253–0.430)	0.345 (0.249–0.457)	Half siblings
KG102/KG121	KGKG	0.372 (0.246–0.452)	0.372 (0.262–0.448)	Half siblings
KG110/KG112	KGKG	0.366 (0.269–0.466)	0.374 (0.279–0.465)	Half siblings
KG104/KG111	KGKG	0.375 (0.283–0.482)	0.381 (0.284–0.482)	Half siblings
KH216/QN214	KHQN	0.356 (0.295–0.489)	0.392 (0.306–0.491)	Half siblings
KG103/KG118	KGKG	0.390 (0.316–0.466)	0.402 (0.320–0.477)	Half siblings
KG104/KG118	KGKG	0.444 (0.350–0.540)	0.447 (0.361–0.54)	Half siblings
KG108/KG204	KGKG	0.610 (0.523–0.694)	0.619 (0.535–0.696)	Full siblings
KG122/KG202	KGKG	0.759 (0.696–0.843)	0.762 (0.696–0.842)	Full siblings
KG107/KG201	KGKG	0.783 (0.721–0.851)	0.785 (0.723–0.851)	Full siblings
PY207/PY209	PYPY	0.842 (0.770–0.892)	0.847 (0.769–0.891)	Full siblings
QN219/QN220	QNQN	0.929 (0.856–0.974)	0.941 (0.869–0.973)	Full siblings

### Population structure and migration patterns

AMOVA results (**Table 3)** of two hierarchical arrangements (3 populations versus 2 populations) and with two data set (with related pairs and related individuals removed) showed the majority of the variation (80.91–87.5%) in *P*. *pelagicus* was found within individuals, and highly significant in all cases (F_IT_ = 0.125–0.19, P<0.001). The proportion of variance explained by differences among populations (F_ST_) were larger in the two-populations (17.43% with related pairs and 15.03% when related individuals removed) than in the three-pops arrangements (11.29% and 8.06%, respectively). It is clear that related individuals contributed to the percentage of variation according to different clustering of populations, however, in all cases the difference were highly significant (P<0.001). With all arrangements and two datasets, among individuals within populations (F_IS_) differentiation were not significant.

**Table 3:** Hierarchical analysis of molecular variance (AMOVA) in *Portunus pelagicus*.

Source of variation	Sum of square	Variant components	% of variation	Fixation index	P value
**Three populations (northern, central and southern) with related pairs**					
Among populations	81.570	0. 580	11.29	F_ST_ = 0.11	<0.001
Among individuals within populations	426.065	0.06	1.21	F_IS_ = 0.01	0.330
Within individuals	428.000	4.46	87.50	F_IT_ = 0.125	<0.001
**Three populations (northern, central and southern) with related individuals removed**					
Among populations	32.271	0. 26	8.06	F_ST_ = 0.08	<0.001
Among individuals within populations	237.742	0.14	4.49	F_IS_ = 0.05	0.07
Within individuals	224.000	2.80	87.45	F_IT_ = 0.125	<0.001
**Two populations (northern–central, southern) with related pairs**					
Among populations	76.98	0. 954	17.43	F_ST_ = 0.174	<0.001
Among individuals within populations	430.690	0. 662	1.13	F_IS_ = 0.01	0.320
Within individuals	428.000	4.46	81.44	F_IT_ = 0.186	<0.001
**Two populations (northern–central, southern) with related individuals removed**					
Among populations	29.708	0. 52	15.03	F_ST_ = 0.15	<0.001
Among individuals within populations	240.305	0. 14	4.06	F_IS_ = 0.05	0.07
Within individuals	224.000	2.80	80.91	F_IT_ = 0.19	<0.001

Pairwise *Fst* values between southern population to northern and central populations showed statistically significant genetic differentiation (P<0.001) in all arrangements, and data sets (**Table 4**). In three-population clustering, the southern population showed more differentiation with the northern (0.199 with related pairs and 0.181 with related individuals removed) than the central (0.143 and 0.117, respectively). However, connectivity was observed between northern and central populations in all cases (Fst = 0.004, P = 0.45 and Fst = 0.0024, P = 0.687).

**Table 4:** Pairwise values of Fst (above the diagonal) and their respective P-values (below the diagonal). Bold values indicate significant differences between populations.

	With related pairs			With related individuals removed		
**Pop ID**	**northern**	**central**	**southern**	**northern**	**central**	**southern**
**northern**	-	0.0004	0.199	-	0.0024	0.181
**central**	0.45	-	0.143	0.687	-	0.117
**southern**	**0.000**	**0.000**	-	**0.000**	**0.000**	-
	**northern-central**		**southern**	**northern-central**		**southern**
**northern-central**	-		0.174	-		0.15
**southern**	**0.000**			**0.000**		

**Fig 3:**
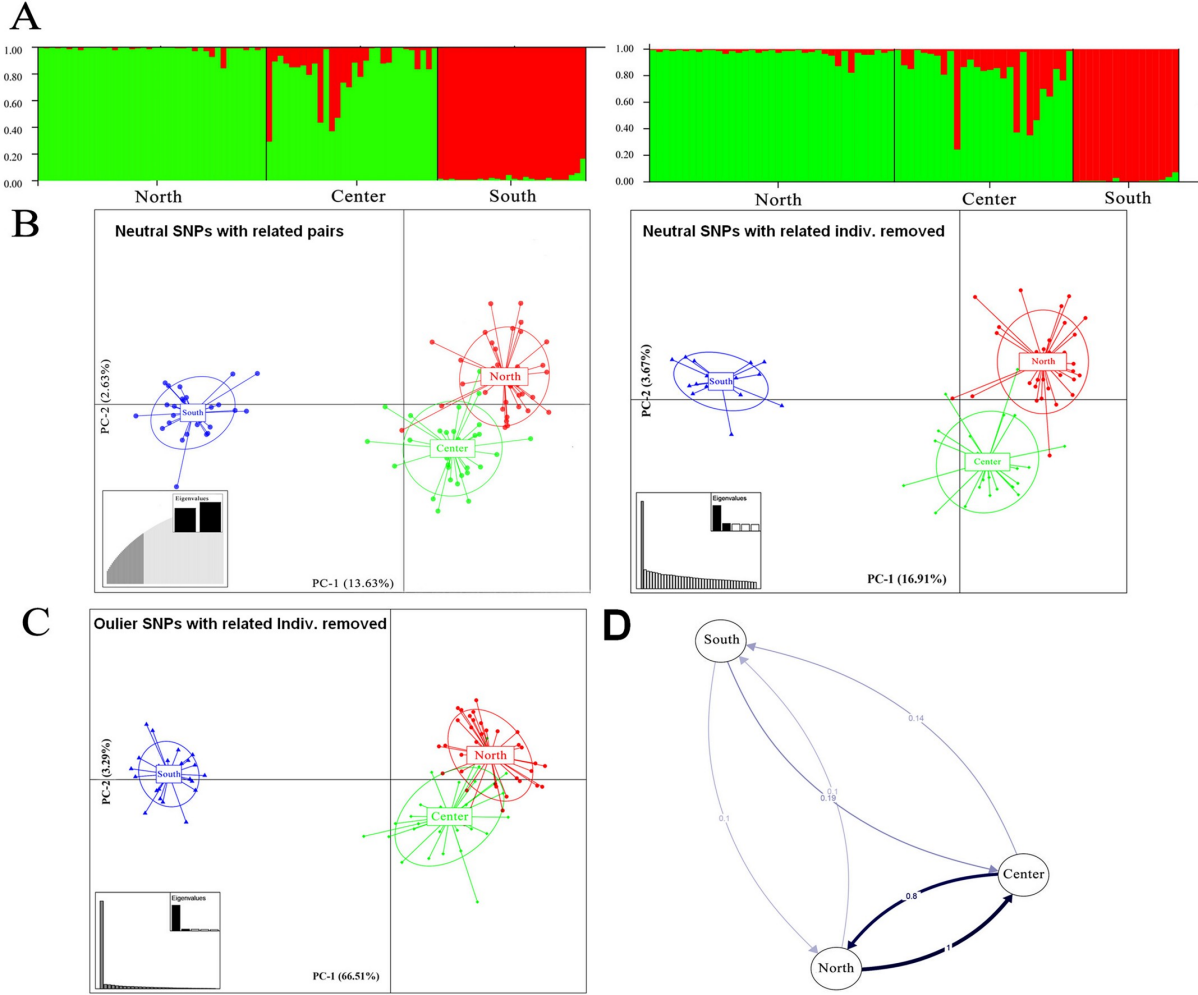
Population structure and migration patterns of *Portunus pelagicus* along the Vietnamese coastline. The bar plot showing individual assignments to inferred clusters (optimal K = 2) using the neutral SNP panels (A) with related pairs (left) and related individuals removed (right) in the program STRUCTURE. Each genotype is represented by a single vertical bar. Scatter plot from DAPC following two neutral SNP panels (B) and outlier loci (C), the percentage of variability explained by each coordinate is shown in brackets. The directional relative migration calculated by the divMigrate function performed in the R package diveRsity (D).

The STRUCTURE analysis, plotted with a *K* of 2 as chosen by the Evanno method, also showed a clear distinction between the south and the remaining two populations. The similar patterns were observed either with related pairs or related individuals removed from SNPs panels. The southern population was assigned to a first lineage with high certainty (98.4% and 98% composition of the “red” lineage and 1.6% and 2% of “green”. Northern and central populations were assigned to the second lineage with the north represented by a dominance (98.2% and 98%) of “green” lineage, and central exhibiting a mixing of “green/red” with percentages of 82.5/17.5 and 80/20 (**Fig 3A left and right**).

The Discriminant analysis of principal component (DAPC) showed a clear distinction between the southern population from northern and central populations in both neutral SNPs data sets (**Fig 3B**). In the dataset with removed related individuals, the northern and central populations were somewhat separated ((**Fig 3B, right**). However, DAPC analysis based on the 32 under-selected loci showed similar results to neutral related pairs SNPs (**Fig 3C**).

Historic migration results strongly supported the Panmixia model based on the highest Bezier approximation score (ln = -115475.9) in which migration was maintained among all sites with random mating between crab individuals (**Table 5**). The analyses disclosed that there was no mating restriction between crab individuals in the history supposed to be over 1000s of years [32,83]. The populations were able to share genetic material either through larval dispersal due to currents or via migration of adult crabs. Directional migration relative rates among recent *P*. *pelagicus* populations range from 0.1 to 1 (**Fig 3D**). Among these, asymmetric directional migration seems to have occurred from southern to northern and central populations, however, bootstrap analysis (nbs<0) showed that directional migration was not significant. Migration from northern to central, however, involved significant asymmetric migration (nbs>0) (**S1 Fig**).

**Table 5:** Log probabilities of the data given the model (marginal likelihood, based on the Bezier approximation score) and Δ values (difference from largest Lm value) and rank according to largest likelihood value.

Model	Bezierln	Delta	Rank
Full	-117538.33	-2062.47	3
South to North	-129937.02	-14461.16	5
North to South	-116622.97	-1147.11	2
**Panmixia**	**-115475.9**	**0**	**1**
South separate	-126655.35	-11179.49	4

## Discussion

The fine scale population structure of swimming crab, applicable in fisheries management was investigated in both putative neutral and outlier loci. Overall, the current analysis based on SNP panels (including or removing related individuals) all showed similar results. The genetic patterns appear to indicate that *P*. *pelagicus* in northern-central and southern areas of the Vietnamese coastline maintain distinct populations. Significant pairwise *F*st comparisons showed strong genetic differentiation between southern to central and northern populations. Furthermore, the hierarchical AMOVA results supported two regional clusters with higher proportions of variation compared to a three-population arrangement (**Table 3**). Structure and DAPC analyses clearly divided the populations into two subdivisions (northern–central and southern). Outlier SNPs, which represented higher genetic differentiation, and respected providing better resolution to detect fine-scale population structure, identified the same patterns as neutral loci, suggested neutral loci themselves may reflected geographical adaptation ([88]. *P*. *pelagicus* is well known as a migratory species, both in adult and larval stages. Male and female crabs can move between estuaries and open oceans for spawning and/or responding to lowered salinities [7]. As spawning of *P*. *pelagicus* occurs year-round [11], following the northeast and southwest monsoons, crab larvae may be dispersed by surface currents (**Fig 1**) along the coast from the Gulf of Tonkin up to the Gulf of Thailand and vice versa. However, all analyses revealed the consistent patterns of non-connectivity from the south to the remaining *P*. *pelagicus* populations.

The Vietnamese coastline in the East Sea is influenced by seasonally complex water circulations, which result in upwelling and anticyclonic/cyclonic eddies along the south and central coasts [3,4,89]. In general, eddies may limit larval dispersal, acting as a larval retention system [90,91] and maintaining divergence in marine populations [92]. Winds, together with tidal and Mekong river discharge (6000–12000 m^3^/s) [93,94] were reported as the factors involved in the upwelling, and separate currents in the southern shelf of Vietnam. That may further well explain restricted gene flow in the southern population. Analyses of contemporary gene flow demonstrated the limited genetic exchange in *P*. *pelagicus* from the south, while the extensive migration occurring along the northern and central coasts. The migration relative rate (Nm) indicated 10 fold greater migration between northern and central populations than from these sites to the south. What makes this more interesting is that significant asymmetric migration from northern to central populations (**S1 Fig**). Monsoon-induced currents and eddies reported in the central coast [3] make the central region a potential population sink. In contrast, estimates of historical gene flow provided strong evidence for a single panmictic population. This may indicate historical patterns of connectivity were different to those detected today. Vietnamese coasts are currently undergoing dramatic changes due to human activities that heavily affect ecosystems and organisms [1,2]. These human induced disturbances such as overexploitation and habitat degradation/fragmentation as well as coastal pollution may prevent larval transport and dispersal by inducing broad-scale larval mortality [33] and obstructing adult migration [28,95], which may be one of the leading causes of current population isolation.

In term of genetic diversity, the lowest value was detected in the southern population, in concordance with high inbreeding coefficient (0.265) as well as related pairs (**Table 2**). This heterozygosity deficiency is also recorded in *P*. *pelagicus* populations in Malaysia [50], and in other marine and freshwater organisms due to widespread habitat loss, degradation and fragmentation [32,96,97]. Significant relatedness and sib-ships have been observed in marine populations due to biophysical larval behavior [98,99], self-recruitment [31,100], and overexploitation/restocking [101]. Kien Giang was the main harvested area of *P*. *pelagicus* in Vietnam, high level of inbreeding and relatedness, and significant genetic differentiation may indicate that local recruitment originates from a limited pool of successful reproductive adults, and reflect somewhat the pressure of overexploitation on crab populations.

This was the first study to apply the powerful technique of over a hundred SNP markers to infer the natural and/or manmade barriers to gene flow in *Portunus pelagicus*. The population structure of *P*. *pelagicus* in the current study does not show high connectivity like other organisms such as lobster [29] and giant clams [53], shown using mitochondrial makers. According to Lemopoulos et al. (2018) [102], RADseq-generate SNPs outperformed microsatellites (and possibly other markers) for investigating individual‐level genotypes, and can be applied to studies of small-scale population structure such as the swimming crab in Vietnam. Looking at the swimming crab in the Indo-Pacific region overall, different patterns and levels of genetic structure of *P*. *pelagicus* have been detected, such as significant genetic differentiation [44,45,47] as well as high gene flow [50,51]. Highly restricted gene flow is mainly reported due to geographic distributions (even at a fine scale) [46], while connectivity is explained by adult migration (such as for spawning), larval dispersal [42], and a lack of physical barriers in the marine environment [50]. In case of Vietnamese *P*. *pelagicus*, the complex natural and anthropogenic biophysical factors may be driving restricted gene flow along the coastline. However, closely related individuals found in the southern population may affect current results such as reducing the sample size (in the case of related individuals removed), or creating an artificial population structure (when included related pairs). However, the two data sets analyses give the same structure, so we can also confirm an accurate reflection of results for a true phenomenon in this species. *P*. *pelagicus* can therefore be considered two fisheries and conservation management units. The factors driving current connectivity patterns of *P*. *pelagicus* are complex, and cannot accurately be identified. *P*. *pelagicus* is likely at risk from inbreeding and subpopulation isolation, and subsequently poor adaptive potential. The management for this species should be careful to ensure that overfishing and habitat degradation do not further affect the vitality of existing populations. Immediate actions such as a seasonal ban on catching crabs in the autumn and late summer to increase successful spawning [32], establishment of marine reserves to reduce genetic losses [101], and coastal pollution control to increase numbers of breeding individuals and larval dispersal [28,33]. Moreover, gear regulation, habitat monitoring and restoration might be one of the most effective ways to manage healthy populations. The appropriate explanation for the high rate of self-recruitment observed in the southern swimming crab remains open. Periodic surveys on genetic diversity, and seascape research [100] should be conducted to provide an overall temporal and spatial view of crab populations.

This study of *P*. *pelagicus* highlighted the important of conservation genetic studies using advanced genomics for information-lacking geographic zones such as Vietnam East Sea. These results also provide important baseline measures of diversity that can be used for future genetic surveys as well as for monitoring responses of *P*. *pelagicus* for environmental changes and temperature rises due to climate change.

## Supporting information

S1 Fig Illustrate the significant directional asymmetric migration calculated by divMigrate function performed in R package diveRsity. Number of bootstraps (nbs) were presented along the links.
(TIF)

S1 Table Sample sites and size of *Portunus pelagicus* with successful sequences, pre-analyzed (*de novo* assembly, mapping) and analyses of population structure. Carapace width (CW) and weight (W). Abbreviation for sampling locations as shown in Table 1.
(DOCX)

S2 Table Numbers of *P*. *pelagicus* individuals and SNPs following the filtering steps, outlier, Linkage disequilibrium analysis and relatedness. (DOCX)

## References

[1] Tran TD. Major issues of coastal environment in Vietnam and orientation for protection In: Hong PN, editor. The role of mangrove and coral reef ecosystems. Publisher: Agriculture Publishing House, Hanoi; 2006 pp. 1–16.

[2] Saito Y, Huy D Van, Tateishi M, Thanh TD, Nguyen VL, Ta TKO. Regimes of human and climate impacts on coastal changes in Vietnam. Reg Environ Chang. 2004;4: 49–62. 10.1007/s10113-003-0062-7

[3] Van Ninh P, Quynh DN, Lanh V Van, Viet Lien N. Geostrophic and drift current in the South China Sea, Area IV : Vietnamese waters. SEAFDEC Semin Fish Resour South Chins Sea, Area IV Vietnamese Waters. 2000; 365–373.

[4] Pasuya MF, Peter BN, MDA H., Omar KM. Sea surface current in the Gulf of Thailand based on nineteen years altimetric data and GPS tracked drifting buoy. Geomatic & Geospatial Technology Conference. 2016 p. 8.

[5] Schmidt-Thomé P, Nguyen TH, Pham TL, Jarva J, Nuottimäki K. Climate change adaptation measures in Vietnam. Springer Briefs Earth Sci. 2015; 7–16. 10.1007/978-3-319-12346-2

[6] Hoegh-Guldberg O, Bruno JF. The impact of climate change on the world’s marine ecosystems. Science (80-). 2010;328: 1523–1528. 10.1126/science.1189930 20558709

[7] Kangas MI. Synopsis of the biology and exploitation of the blue swimmer crab, *Portunus pelagicus* Linnaeus, in Western Australia. Fish. Res. Rep. Fish. West. Aust. 2000.

[8] Banks R, Banks R, Holt T. Assessment report for Vietnamese: Blue swimming crab tangle net fishery (*Portunus pelagicus*), Kien Giang province. 2009.

[9] Kunsook C, Gajaseni N, Paphavasit N. A stock assessment of the blue swimming crab *Portunus pelagicus* (Linnaeus, 1758) for sustainable management in Kung Krabaen Bay, Gulf of Thailand. Trop Life Sci Res. 2014;25: 41–59. PMC4156473 25210587

[10] Lai JCY, Ng PKL, Davie PJF. A revision of the *Portunus pelagicu*s (Linnaeus, 1758) species complex (Crustacea: Brachyura: Portunidae), with the recognition of four species. Raffles Bull Zool. 2010;58: 199–237.

[11] Ha VV, Nhan TH, Cuong T Van, Doan NS. Stock and fishery assessment report of blue swimming crab P*ortunus pelagicus* (Linnaeus, 1758) in Kien Giang waters, Viet Nam. Report for WWF and WASEP. 2014.

[12] Canan T, Irem Y. Reproductive biology of the blue swimming crab, *Portunus segnis* (Forskal, 1775) in Yumurtalık Cove, North-eastern Mediterranean, Turkey. Mediterr Mar Sci. 2017;18: 424–432. 10.12681/mms.13789

[13] Ernawati TRI, Sumiono B, Madduppa H. Reproductive ecology, spawning potential, and breeding season of blue swimming crab (Portunidae: *Portunus pelagicu*s) in Java Sea, Indonesia. Biodiversitas. 2017;18: 1705–1713. 10.13057/biodiv/d180451

[14] Zairion, Wardiatno Y, Fahrudin A. Sexual maturity, reproductive pattern and spawning female population of the blue swimming crab, *Portunus pelagicus* (Brachyura: Portunidae) in East Lampung Coastal Waters, Indonesia. Indian J Sci Technol. 2015;8: 596 10.17485/ijst/2015/v8i6/69368 PMC4437326 26019748

[15] Bryars SR, Havenhand JN. Temporal and spatial distribution and abundance of blue swimmer crab (*Portunus pelagicus*) larvae in a temperate gulf. Mar Freshw Res. 2004;55: 809–818. 10.1071/MF04045

[16] Bryars S. Larval Dispersal of the Blue Swimmer Crab, *Portunus Pelagicus* (Linnaeus) (Crustacea:Decapoda: Portunidae), in South Australia. Flinders University of South Australia 1997.

[17] Kemnaren DD, Zarion, Kamal MM, Wardiatno Y. Abundance and spatial distribution of blue swimming crab (*Portunus pelagicus*) larvae during east monsoon in the East Lampung waters, Indonesia. Biodiversitas J Biol Divers. 2018;19: 1326–1333. 10.13057/biodiv/d190420

[18] Macale AMB, Alcantara SG, Nieves PM. Density distribution of blue crab (*Portunus pelagicus*) larvae with implications to the lying-in concept of stock enhancement. Kuroshio Sci. 2017;11: 54–62.

[19] Svane I, Hooper G. Blue Swimmer Crab (*Portunus pelagicus*) Fishery. Fishery Assessment Report to PIRSA for the Blue Crab Fishery Management Committee 2004.

[20] VASEP. Vietnam is the leading supplier of fresh blue swimming crab to Japan. Vietnam Association of Seafood Exporters and Producers report, 09/2013. [http://www.seafood.vasep.com.vn/Daily-News/378_8240/Vietnam-is-the-leading-supplier-of-fresh-blue-swimming-crab-to-Japan.htm]. 2013.

[21] FAO. Species Fact Sheets *Portunus pelagicus* (Linnaeus, 1758) Fisheries. Fisheries and Aquaculture Department 2016.

[22] Ha VV, Nhan TH, Cuong TV, Doan NS. Stock and fishery assessment report of blue swimming crab *Portunus pelagicus* (Linnaeus, 1758) in Kien Giang waters, Viet Nam. 2015.

[23] Seafood Watch Consulting Researcher. Blue swimming crab Vietnam and Gulf of Thailand Bottom gillnet, Pots, Set gillnet, Traps. 2018.

[24] Berger AM, Goethel DR, Lynch PD, Quinn II T, Mormede S, McKenzie J, et al Space oddity: The mission for spatial integration. Can J Fish Aquat Sci. 2017;74: 1698–1716. 10.1139/cjfas-2017-0150

[25] Kerr LA, Hintzen NT, Cadrin SX, Clausen LW, Dickey-Collas M, Goethel DR, et al Lessons learned from practical approaches to reconcile mismatches between biological population structure and stock units of marine fish. ICES J Mar Sci. 2017;74: 1708–1722. 10.1093/icesjms/fsw188

[26] Kritzer JP, Liu OR. Fishery management strategies for addressing complex spatial structure in marine fish stocks In: Cadrin S. X., Kerr SM L. A., editor. Stock identification methods: Applications in Ffshery science. Second Edi Academic Press, San Diego, California; 2014 pp. 29–57. 10.1016/B978-0-12-397003-9.00003-5

[27] Reiss H, Hoarau G, Dickey-Collas M, Wolff WJ. Genetic population structure of marine fish: Mismatch between biological and fisheries management units. Fish Fish. 2009;10: 361–395. 10.1111/j.1467-2979.2008.00324.x

[28] Cimmaruta R, Scialanca F, Luccioli F, Nascetti G. Genetic diversity and environmental stress in Italian populations of the cyprinodont fish *Aphanius fasciatus*. Oceanol Acta. 2003;26: 101–110. 10.1016/S0399-1784(02)01234-3

[29] Dao HT, Smith-keune C, Wolanski E, Jones CM. Oceanographic currents and local ecological knowledge indicate, and genetics does not refute, a Contemporary pattern of larval dispersal for The ornate spiny lobster, *Panulirus ornatus* in the South-East Asian Archipelago. PLoS One. 2015;10: 1–19. 110.1371/journal.pone.0124568 10.1371/journal.pone.0124568 PMC4423998 25951344

[30] Dang BT, Vu QHD, Biesack EE, Doan T V., Truong OT, Tran TL, et al Population genomics of the peripheral freshwater fish *Polynemus melanochir* (Perciformes, Polynemidae) in a changing Mekong Delta. Conserv Genet. 2019; 10.1007/s10592-019-01157-5

[31] Truelove NK, Kough AS, Behringer DC, Paris CB, Box SJ, Preziosi RF, et al Biophysical connectivity explains population genetic structure in a highly dispersive marine species. Coral Reefs. Springer Berlin Heidelberg; 2017;36: 233–244. 10.1007/s00338-016-1516-y

[32] Nehemia A, Kochzius M. Reduced genetic diversity and alteration of gene flow in a fiddler crab due to mangrove degradation. PLoS One. 2017;12: 1–20. 10.1371/journal.pone.0182987 PMC5570428 28837577

[33] Puritz JB, Toonen RJ. Coastal pollution limits pelagic larval dispersal. Nat Commun. Nature Publishing Group; 2011;2: 226–228. 10.1038/ncomms1238 21407192

[34] Hamilton PB, Cowx IG, Oleksiak MF, Griffiths AM, Grahn M, Stevens JR, et al Population-level consequences for wild fish exposed to sublethal concentrations of chemicals–a critical review. FFish Fish. 2016;17: 545–566. 10.1111/faf.12125

[35] Kenchington EL. The effects of fishing on species and genetic diversity In: Sinclair M, Valdimarson G, editors. Responsible fisheries in the marine ecosystem. CAB International, Wallingford, Oxon, UK; 2003 pp. 253–272. 10.1079/9780851996332.0235

[36] Smith P. Genetic diversity of marine fisheries resources: possible impacts of fishing. FAO Fisheries Technical paper No. 344. Rome, FAO. 1994.

[37] Hutchings JA RJ. Marine fish population collapses: consequences for recovery and extinction risk. Bioscience. 2004;54: 297–309. 10.1641/0006-3568(2004)054[0297:MFPCCF]2.0.CO;2

[38] Peterson BK, Weber JN, Kay EH, Fisher HS, Hoekstra HE. Double digest RADseq: An inexpensive method for de novo SNP discovery and genotyping in model and non-model species. PLoS One. 2012;7 10.1371/journal.pone.0037135 PMC3365034 22675423

[39] Al-Breiki RD, Kjeldsen SR, Afzal H, Al Hinai MS, Zenger KR, Jerry DR, et al Genome-wide SNP analyses reveal high gene flow and signatures of local adaptation among the scalloped spiny lobster (*Panulirus homarus*) along the Omani coastline. BMC Genomics. BMC Genomics; 2018;19: 1DUMMY 10.1186/s12864-018-5044-8 30231936 PMC6146514

[40] Vigouroux R, Roussel J-M, Lassalle G, Longin G, Rinaldo R, Barloy D, et al A cost-and-time effective procedure to develop SNP markers for multiple species: A support for community genetics. Methods Ecol Evol. 2018;9: 1959–1974. 10.1111/2041-210x.13034

[41] Etter PD, Selker EU, Currey MC, Cresko WA, Baird NA, Atwood TS, et al Rapid SNP discovery and genetic mapping using sequenced RAD markers. PLoS One. 2008;3: e3376 10.1371/journal.pone.0003376 18852878 PMC2557064

[42] Wang S, Meyer E, Mckay JK, Matz M V. 2b-RAD: A simple and flexible method for genome-wide genotyping. Nat Methods. 2012;9: 808–810. 10.1038/nmeth.2023 22609625

[43] Bird CE, Andrews KR, Fernandez-Silva I, Forsman ZH, Toonen RJ, Puritz JB, et al ezRAD: a simplified method for genomic genotyping in non-model organisms. PeerJ. 2013;1: e203 10.7717/peerj.203 24282669 PMC3840413

[44] Yap ES, Sezmis E, Chaplin JA, Potter IC, Spencer PBS. Isolation and characterization of microsatellite loci in *Portunus pelagicus* (Crustacea: Portunidae). Mol Ecol Notes. 2002;2: 30–32. 10.1046/j.1471-8286.2002.00136.x

[45] Sezmiş E. The population genetic structure of *Portunus pelagicus* in Australian waters. Ph.D thesis, Murdoch University, Australia. 2004.

[46] Klinbunga S, Khetpu K, Khamnamtong B, Menasveta P. Genetic heterogeneity of the blue swimming crab (*Portunus pelagicus*) in Thailand determined by AFLP analysis. Biochem Genet. 2007;45: 725–736. 10.1007/s10528-007-9110-1 17879155

[47] Klinbunga S, Yuvanatemiya V, Wongphayak S, Khetpu K, Menasveta P, Khamnamtong B. Genetic population differentiation of the blue swimming crab *Portunus pelagicus* (Portunidae) in Thai waters revealed by RAPD analysis. Genet Mol Res. 2010;9: 1615–1624. 10.4238/vol9-3gmr886 20730713

[48] Ren G, Miao G, Ma C, Lu J, Yang X, Ma H. Genetic structure and historical demography of the blue swimming crab (*Portunus pelagicus*) from southeastern sea of China based on mitochondrial COI gene. Mitochondrial DNA Part A DNA Mapping, Seq Anal. Informa UK Ltd.; 2018;29: 192–198. 10.1080/24701394.2016.1261855 28034343

[49] Sienes PMQ, Willette DA, Romena LR, Alvior CG, Estacion JS, Malay MCD. Genetic diversity and the discovery of a putative cryptic species within a valued crab fishery, *Portunus pelagicus* (Linnaeus 1758), in the Philippines. Philipp Sci Lett. 2014;7: 317–323.

[50] Chai CJ, Esa Y Bin, Ismail MFS, Kamarudin MS. Population structure of *Portunus pelagicus* in coastal areas of Malaysia inferred from microsatellites. Zool Stud. 2017;56: 1–12. 10.6620/ZS.2017.56-26 PMC6517759 31966225

[51] Andi IA, Andi T, Andi AH, Yushinta F, Andi P. High genetic variation of *Portunus pelagicus* from Makassar Straits revealed by RAPD markers and mitochondrial 16S rRNA sequences. African J Biotechnol. 2016;15: 180–190. 10.5897/ajb2015.15045

[52] Yang X, You C, Wang S, Miao G, Shi X, Wu Q, et al Isolation and characterization of 91 single nucleotide polymorphism (SNP) markers for the blue swimming crab (*Portunus pelagicus*). Conserv Genet Resour. Springer Netherlands; 2017;9: 549–556. 10.1007/s12686-017-0720-6

[53] Nguyen TAT, Dang TB, Chau TML. A study of genetic structure of giant clam (*Tridacna* spp.) (Tridacninae) population in south central and southern Vietnam’s coast. J Biol. 2014;36 (1se): 189–194.

[54] Puritz JB, Hollenbeck CM, Gold JR. dDocent : a RADseq, variant-calling pipeline designed for population genomics of non-model organisms. PeerJ. 2014;2: e431 10.7717/peerj.431 24949246 PMC4060032

[55] Bolger AM, Lohse M, Usadel B. Trimmomatic: A flexible trimmer for Illumina sequence data. Bioinformatics. 2014;30: 2114–2120. 10.1093/bioinformatics/btu170 24695404 PMC4103590

[56] Chong Z, Ruan J, Wu CI. Rainbow: An integrated tool for efficient clustering and assembling RAD-seq reads. Bioinformatics. 2012;28: 2732–2737. 10.1093/bioinformatics/bts482 22942077

[57] Fu L, Niu B, Zhu Z, Wu S, Li W. CD-HIT: Accelerated for clustering the next-generation sequencing data. Bioinformatics. 2012;28: 3150–3152. 10.1093/bioinformatics/bts565 23060610 PMC3516142

[58] Li W, Godzik A. Cd-hit: A fast program for clustering and comparing large sets of protein or nucleotide sequences. Bioinformatics. 2006;22: 1658–1659. 10.1093/bioinformatics/btl158 16731699

[59] Li H, Durbin R. Fast and accurate long-read alignment with Burrows-Wheeler transform. Bioinformatics. 2009;25: 1754–1760. 10.1093/bioinformatics/btp324 19451168 PMC2705234

[60] Li H, Durbin R. Fast and accurate long-read alignment with Burrows-Wheeler transform. Bioinformatics. 2010;26: 589–595. 10.1093/bioinformatics/btp698 20080505 PMC2828108

[61] Li H. Aligning sequence reads, clone sequences and assembly contigs with BWA-MEM. Oxford Univ Press 2013 2013;00: 1–3. 10.1186/s13756-018-0352-y

[62] Wysoker A, Fennell T, Marth G, Abecasis G, Ruan J, Li H, et al The Sequence Alignment/Map format and SAMtools. Bioinformatics. 2009;25: 2078–2079. 10.1093/bioinformatics/btp352 19505943 PMC2723002

[63] Garrison E, Marth G. Haplotype-based variant detection from short-read sequencing [Internet]. 2012 pp. 1–9. arXiv:1207.3907

[64] Banks E, Lunter G, Albers CA, Durbin R, Danecek P, Auton A, et al The variant call format and VCFtools. Bioinformatics. 2011;27: 2156–2158. 10.1093/bioinformatics/btr330 21653522 PMC3137218

[65] Foll M, Gaggiotti O. A genome-scan method to identify selected loci appropriate for both dominant and codominant markers: A Bayesian perspective. Genetics. 2008;180: 977–993. 10.1534/genetics.108.092221 18780740 PMC2567396

[66] Benajmini Y, Hochberg Y, Benjamini Y, Hochberg Y. Controlling the False Discovery Rate : A Practical and powerful approach to Multiple Testing. J R Stat Soc B. 1995;57: 289–300. 10.2307/2346101

[67] Gregory W., Gregor G. FL and MM. Genetics: Population Genetics. R package version 1.3.8.1.1 [Internet]. 2019. Available: https://cran.r-project.org/package=genetics

[68] Kemppainen P, Hlaing T, Walton C, Somboon P, Knight CG, Mahanta J, et al Linkage disequilibrium network analysis (LDna) gives a global view of chromosomal inversions, local adaptation and geographic structure. Mol Ecol Resour. 2015;15: 1031–1045. 10.1111/1755-0998.12369 25573196 PMC4681347

[69] Csardi G. NT. The igraph software package for complex network research, InterJournal, Complex Systems 1695. http://igraph.org; 2006.

[70] Peakall R, Smouse PE. GenALEx 6.5: Genetic analysis in Excel. Population genetic software for teaching and research-an update. Bioinformatics. 2012;28: 2537–2539. 10.1093/bioinformatics/bts460 22820204 PMC3463245

[71] Meirmans Patrick G., Tienderen VH. GENOTYPE and GENODIVE : two programs for the analysis of genetic diversity of asexual organisms. Mol Ecol Notes. 2004;4: 792–794. 10.1111/j.1471-8286.2004.00770.x

[72] Pew J, Muir PH, Wang J, Frasier TR. related: An R package for analysing pairwise relatedness from codominant molecular markers. Mol Ecol Resour. 2015;15: 557–561. 10.1111/1755-0998.12323 25186958

[73] Milligan BG. Maximum-likelihood estimation of relatedness. Genetics. 2003;163: 1153–1167. 10.4289/0013-8797-112.1.1 12663552 PMC1462494

[74] Wang J. Triadic IBD coefficients and applications to estimating pairwise relatedness. Genet Res. 2007;89: 135–153. 10.1017/S0016672307008798 17894908

[75] Waples RS, Anderson EC. Purging putative siblings from population genetic data sets: A cautionary view. Mol Ecol. 2017;26: 1211–1224. 10.1111/mec.14022 28099771

[76] Excoffier L, Laval G, Schneider S. Arlequin (version 3.0): An integrated software package for population genetics data analysis. Evol Bioinforma. 2017;1: 117693430500100 10.1177/117693430500100003 PMC2658868 19325852

[77] Hubisz MJ, Falush D, Stephens M, Pritchard JK. Inferring weak population structure with the assistance of sample group information. Mol Ecol Resour. 2009;9: 1322–1332. 10.1111/j.1755-0998.2009.02591.x 21564903 PMC3518025

[78] Daniel Falush MS and JKP. Inference of population structure using multilocus genotype data: Linked loci and correlated allele frequencies. Genetics. 2003;164: 1567–1587. 12930761 10.1093/genetics/164.4.1567 PMC1462648

[79] Evanno G, Regnaut S, Goudet J. Detecting the number of clusters of individuals using the software STRUCTURE: A simulation study. Mol Ecol. 2005;14: 2611–2620. 10.1111/j.1365-294X.2005.02553.x 15969739

[80] Earl DA, vonHoldt BM. STRUCTURE HARVESTER: A website and program for visualizing STRUCTURE output and implementing the Evanno method. Conserv Genet Resour. 2012;4: 359–361. 10.1007/s12686-011-9548-7

[81] Jombart T, Ahmed I. adegenet 1.3–1: New tools for the analysis of genome-wide SNP data. Bioinformatics. 2011;27: 3070–3071. 10.1093/bioinformatics/btr521 21926124 PMC3198581

[82] Beerli P, Felsenstein J. Maximum likelihood estimation of a migration matrix and effective population sizes in n subpopulations by using a coalescent approach. Proc Natl Acad Sci USA. 2001;98: 4563–456. 10.1073/pnas.081068098 11287657 PMC31874

[83] Chiucchi JE, Gibbs HL. Similarity of contemporary and historical gene flow among highly fragmented populations of an endangered rattlesnake. Mol Ecol. 2010;19: 5345–5358. 10.1111/j.1365-294X.2010.04860.x 20964755

[84] Beerli P. How to use M IGRATE or why are Markov chain Monte Carlo programs difficult to use? Bertorelle G, Bruford MW, Hauffe HC, Rizzoli A, Vernesi (eds) Population genetics for animal conservation. The Cambridge University Press, Cambridge; 2009 pp. 42–79.

[85] Sundqvist L, Keenan K, Zackrisson M, Prodöhl P, Kleinhans D. Directional genetic differentiation and relative migration. Ecol Evol. 2016;6: 3461–3475. 10.1002/ece3.2096 27127613 PMC4842207

[86] Keenan K., McGinnity P., Cross T.F., Crozier W.W., & Prodöhl PA. DiveRsity: An R package for the estimation of population genetics parameters and their associated errors. Methods Ecol Evol. https://CRAN.R-project.org/package=genetics; 2013;4: 782–788. 10.1111/2041-210X.12067

[87] Epskamp S, Cramer AOJ, Waldorp LJ, Schmittmann VD, Borsboom D. qgraph : Network visualizations of relationships in psychometric data. J Stat Softw. 2012;48: 1–18. 10.18637/jss.v048.i04

[88] Moore JS, Bourret V, Dionne M, Bradbury I, O’Reilly P, Kent M, et al Conservation genomics of anadromous Atlantic salmon across its North American range: Outlier loci identify the same patterns of population structure as neutral loci. Mol Ecol. 2014;23: 5680–5697. 10.1111/mec.12972 25327895

[89] Chen C, Lai Z, Beardsley RC, Xu Q, Lin H, Viet NT. Current separation and upwelling over the southeast shelf of Vietnam in the South China Sea. J Geophys Res. 2012;117: C03033 10.1029/2011JC007150

[90] Morgan SG, Fisher JL, Miller SH, McAfee ST, Largier JL. Nearshore larval retention in a region of strong upwelling and recruitment limitation. Ecology. 2009;90: 3489–3502. 10.1890/08-1550.1 20120816

[91] Condie S, Condie R. Retention of plankton within ocean eddies. Glob Ecol Biogeogr. 2016;25: 1264–1277. 10.1111/geb.12485

[92] Pineda J, Hare J, Sponaugle S. Larval transport and dispersal in the coastal ocean and consequences for population connectivity. Oceanography. 2011;20: 22–39. 10.5670/oceanog.2007.27

[93] Hung NN, Delgado JM, Tri VK, Hung LM, Merz B, Bárdossy A, et al Floodplain hydrology of the mekong delta, Vietnam. Hydrol Process. 2012;26: 674–686. 10.1002/hyp.8183

[94] Tri VK. Hydrology and hydraulic infrastructure systems in the Mekong Delta, Vietnam Renaud F, Kuenzer C The Mekong Delta System Springer Environmental Science and Engineering. Dordrecht: Springer; 2012 pp. 49–81. 10.1007/978-94-007-39-62_3

[95] Gonzalez EB, Knutsen H, Jorde PE. Habitat discontinuities separate genetically divergent populations of a rocky shore marine fish. PLoS One. 2016;11 10.5061/dryad.4g349.Funding PMC5051803 27706178

[96] Yuhara T, Kawane M, Furota T. Genetic population structure of local populations of the endangered saltmarsh sesarmid crab *Clistocoeloma sinense* in Japan. PLoS One. 2014;9: 1–9. 10.1371/journal.pone.0084720 PMC3882244 24400112

[97] Ackiss AS, Dang BT, Bird CE, Biesack EE, Chheng P, Phounvisouk L, et al Cryptic lineages and a population dammed to incipient extinction? Insights into the genetic structure of a Mekong River catfish. J Hered. 535–547. 10.1093/jhered/esz016 30887034

[98] Aglieri G, Papetti C, Zane L, Milisenda G, Boero F, Piraino S. First evidence of inbreeding, relatedness and chaotic genetic patchiness in the holoplanktonic jellyfish *Pelagia noctiluca* (Scyphozoa, Cnidaria). PLoS One. 2014;9 10.1371/journal.pone.0099647 PMC4076186 24977703

[99] Riesgo A, Taboada S, Pérez-Portela R, Melis P, Xavier JR, Blasco G, et al Genetic diversity, connectivity and gene flow along the distribution of the emblematic Atlanto-Mediterranean sponge *Petrosia ficiformis* (Haplosclerida, Demospongiae). BMC Evol Biol. BMC Evolutionary Biology; 2019;19: 1–18. 10.1186/s12862-018-1333-8 30651060 PMC6335727

[100] Teske PR, Sandoval-Castillo J, Van Sebille E, Waters J, Beheregaray LB. Oceanography promotes self-recruitment in a planktonic larval disperser. Sci Rep. Nature Publishing Group; 2016;6: 1–8. 10.1038/s41598-016-0001-8 27687507 PMC5043232

[101] Munguía-Vega A, Sáenz-Arroyo A, Greenley AP, Espinoza-Montes JA, Palumbi SR, Rossetto M, et al Marine reserves help preserve genetic diversity after impacts derived from climate variability: Lessons from the pink abalone in Baja California. Glob Ecol Conserv. Elsevier B.V.; 2015;4: 264–276. 10.1016/j.gecco.2015.07.005

[102] Lemopoulos A, Prokkola JM, Uusi-Heikkilä S, Vasemägi A, Huusko A, Hyvärinen P, et al Comparing RADseq and microsatellites for estimating genetic diversity and relatedness—Implications for brown trout conservation. Ecol Evol. 2019;9: 2106–2120. 10.1002/ece3.4905 30847096 PMC6392366

