# Supplementary material for: Genome-wide SNP analyses reveal population structure of Portunus pelagicus along Vietnam coastline
Source: PLoS One. 2019 Nov 5;14(11):e0224473. doi: 10.1371/journal.pone.0224473 (PMC6830773; doi:10.1371/journal.pone.0224473)
Supplement: S1 Table — Carapace width (CW) and weight (W). Abbreviation for sampling locations as shown in Table 1. (DOCX) [file pone.0224473.s002.docx]

**S1 Table:** **Sample sites and size of *Portunus pelagicus*** with successful sequences, pre-analyzed (*de novo* assembly, mapping) and analyses of population structure. Carapace width (CW) and weight (W). Abbreviation for sampling locations as shown in Table 1

| **Sites** | **Geographic coordinates** | | **Sequenced** | **Size** | | **Pre-analyzed** | **Analyzed** |
| --- | --- | --- | --- | --- | --- | --- | --- |
|  |  |  |  | **CW** | **W** |  |  |
| HP | 20° 51′ N | 106° 41′ E | 41 | 9.52±0.97 | 94.13±32.54 | 23 | 16 |
| QN | 21° 15′ N | 107° 20′ E | 24 | 9.52±0.97 | 94.13±32.54 | 24 | 24 |
| KH | 12° 15′ N | 109° 12′ E | 37 | 9.47±1.57 | 95.81±30.83 | 23 | 19 |
| PY | 13° 10′ N | 109° 10′ E | 35 | 9.62±1.76 | 86.26±34.4 | 11 | 11 |
| PQ | 10° 14′ N | 103° 57′ E | 18 | 10.36±1.2 | 99.36±25.76 | 18 | 16 |
| RG | 10° 1′ N | 105° 5′ E | 10 | 9.6±1.43 | 82.9±11.53 | 10 | 10 |
| Overall | | | 165 | 10.46±2.0 | 110.27±47.5 | 109 | 96 |
