# Supplementary material for: Genome-wide SNP analyses reveal population structure of Portunus pelagicus along Vietnam coastline
Source: PLoS One. 2019 Nov 5;14(11):e0224473. doi: 10.1371/journal.pone.0224473 (PMC6830773; doi:10.1371/journal.pone.0224473)
Supplement: S2 Table — (DOCX) [file pone.0224473.s003.docx]

**S2 Table: Numbers of *P. pelagicus*** i**ndividuals and SNPs following the filtering steps, outlier, Linkage disequilibrium analysis and relatedness**

| Filtering steps | No of Individuals | No of SNPs |
| --- | --- | --- |
| MAF > 0.05 | 96 | 36609 |
| 5 <= meandp <= 10 | 96 | 4857 |
| Remove-indel | 96 | 3161 |
| HWE < 0.001 | 96 | 3156 |
| MinQ > 30 | 96 | 2923 |
| Max-missing 0.9 | 96 | 1240 |
| MAC 3 | 96 | 1240 |
| AB | 96 | 817 |
| MQM | 96 | 623 |
| PAIRED | 96 | 606 |
| RAD_HAPLOTYPES | 96 | 423 |
| Filter_one_SNPs | 96 | 338 |
| Outlier and LD analysis | 96 | 306 |
| Relatedness | 80 | 306 |
